# Resilience mediates the prediction of alienation towards parents on subjective well-being in rural left-behind children: a 12-month longitudinal study

**DOI:** 10.3389/fpsyt.2024.1414575

**Published:** 2024-07-09

**Authors:** Mengjia Zhang, Xiaoxiao Sun, Chun Cui, Jieying Tan, Xiaomei Ren, Qin Dai

**Affiliations:** ^1^ Department of Medical Psychology, Army Medical University, Chongqing, China; ^2^ The third Recuperation Department of Naval Special Servicemen Recuperation Center of People's Liberation Army, Qingdao, China; ^3^ Radiological Department, The Xinqiao Hospital of the Army Medical University, Chongqing, China; ^4^ School of Public Policy and Administration, Chongqing University, Chongqing, China; ^5^ Southwest Hospital, Army Medical University, Chongqing, China

**Keywords:** alienation towards parents, left-behind children (LBC), subjective well-being, resilience, hierarchical linear modeling (HLM)

## Abstract

**Objective:**

Parent-child relationship is critical for children’s well-being. In China, the large number of left-behind children (LBC, one or both parents leaving for work for at least 6 months) raises public concern. Although LBC often report poor mental health status and higher alienation towards parents, the dynamic trend of subjective well-being in this population, as well as the prediction of alienation towards parents on LBC’s subjective well-being, remain unrevealed. This study aimed to examine the dynamic trend of subjective well-being in Chinese LBC and further explore the predictional influence of alienation towards parents, with resilience as a potential mediator.

**Methods:**

We recruited 916 rural LBC in China and investigated them at five waves (baseline, and 1, 3, 6, and 12 months later) using Inventory of Alienation towards Parents (IAP), Resilience Scale for Chinese Adolescents (RSCA) and Subjective Happiness Scale (SHS). We used hierarchical linear modeling (HLM) for analysis.

**Results:**

At baseline, no significant differences were found in the scores of alienation towards parents, resilience, and subjective well-being on gender, grade, or type of LBC. A significant correlation existed between the scores of alienation towards parents, resilience, and subjective well-being. HLM showed a linear increase in the subjective well-being of rural LBC. Alienation toward both mother and father negatively predicted the developmental trajectory of children’s subjective well-being over 12 months. Moreover, resilience partially mediated this prediction.

**Conclusion:**

This study is among the first to reveal that alienation towards parents predicts the developmental trajectory of later LBC’s subjective well-being, with resilience as a mediator. These findings warrant the necessity of paying attention to alienation toward parents to ensure the mental health of LBC, giving valuable guidance to parents, schools and governments.

## 1 Introduction

It is well known that parent-child relationship is critical for children’s well-being (1). In China, rural parents go out to pursue a better life, significantly increasing population mobility. Millions of children are left-behind due to constraints on migrated workers, such as limited housing, children’s educational policies and opportunities, and household registration (Hukou) restrictions (2). Left behind children (LBC) refer to children who are left at home with one or both parents leaving home for work for at least six months (3). A 2020 survey by the Chinese Academy of Social Sciences reported more than 66.93 million LBC in China, accounting for 62.4% of all children in rural areas and 23.23% of young people in the whole country, an increase of 12.02 million since 2010 (3–5). The Chinese State Council has pointed out that “to strengthen the care and protection of LBC in rural areas and create a better environment for them is a primary and urgent task of government.” Notably, Chinese LBC reported more emotional problems and poor behavioral functions due to parental absence (6–8). Thus, to ensure the growth of LBC, their mental health status cannot be disregarded.

For children, subjective well-being represents mental health well, reflecting their overall emotional and cognitive perception of their life quality (9). Researchers point out that increasing subjective well-being is one of the primary objectives of improving mental health (10). However, the subjective well-being level of LBC is typically lower than non-LBC (11–14). Most existing studies on LBC were cross-sectional (11–14), ignoring the dynamics of subjective well-being. Thus, the multi-time-point survey (a longitudinal study) on LBC’s subjective well-being remains unrevealed, which is important to reflect the trend of mental health status in this population.

Numerous factors may influence the LBC’s subjective well-being, with the parent-child relationship likely being the most significant (1). Previous studies have used indicators that reflect the solid emotional bond between children and parents (15), such as parent-child attachment and cohesiveness to gauge the quality of parent-child relationships (16). However, the degree of intimacy and alienation can also reflect the parents-child relationship. Alienation towards parents is the expression of children’s unfavorable emotions, such as emotional aloofness, possessiveness, or even being under control when interacting with parents (17, 18). Compared with the non-LBC, the LBC tend to communicate less with their parents and report a stronger sense of alienation towards them (19, 20). Moreover, children with a strong sense of alienation were prone to psychosomatic health problems, such as depression, sleep and eating disorders, and even suicidal ideation (21). Despite these reports on the well-being of children, the direct impact of alienation towards parents on children’s subjective well-being remains unknown. Furthermore, the prediction of alienation on LBC’s subjective well-being remains unaddressed, which is potentially important to reveal the mechanism behind LBC’s well-being fully.

Moreover, the influence of alienation towards parents on LBC’s subjective well-being may have a mediating pathway. Resilience represents desirable adaptation competence when suffering from adverse experiences (22). It has been revealed to minimize individuals’ psychological and physiological costs in coping with adversity (23) and directly promote psychological well-being, such as happiness and life-satisfaction (24, 25). Previous investigations have further indicated that LBC with poor parent-child bonds showed significantly lower psychological resilience (26). The results indicated a connection between alienation towards parents, poor subjective well-being, and lower resilience in children. Although a study has demonstrated that family support can partially maintain mental strength and well-being through resilience in adults (27), it remains uncovered whether resilience mediates the effect of alienation towards parents on subjective well-being of children. Clarifying this potential mediating role is important to reveal the underlying mechanism of subjective well-being in LBC.

Given the research context described above, we propose the following hypotheses:

1. Subjective well-being in LBC may vary across time.
2. Alienation towards parents may negatively predict LBC’s subjective well-being 12 months later.
3. Resilience may mediate the impact of alienation towards parents on LBC’s subjective well-being.

## 2 Methods

### 2.1 Participants

The present study utilized five waves of data collected across 12 months from LBC attending three rural primary schools in Chongqing City, southwestern China. LBC from these schools were invited to participate in this longitudinal survey. The inclusion criteria were: (1) being in grades 4–6, (2) having guardians’ consent to participate, and (3) personally agreeing to participate.

Children were classified as left-behind if (1) either parent had left for work over the past six months and (2) he/she currently lives with one parent, grandparents, relatives, or other caregivers. The LBC were further divided into subgroups: (1) “mother absent”: only the mother had left for work, (2)”father absent”: only the father has left for work, and (3)”both absent”: both parents had left for work. We excluded children from single-parent families to focus on left-behind status.

**Figure 1:**
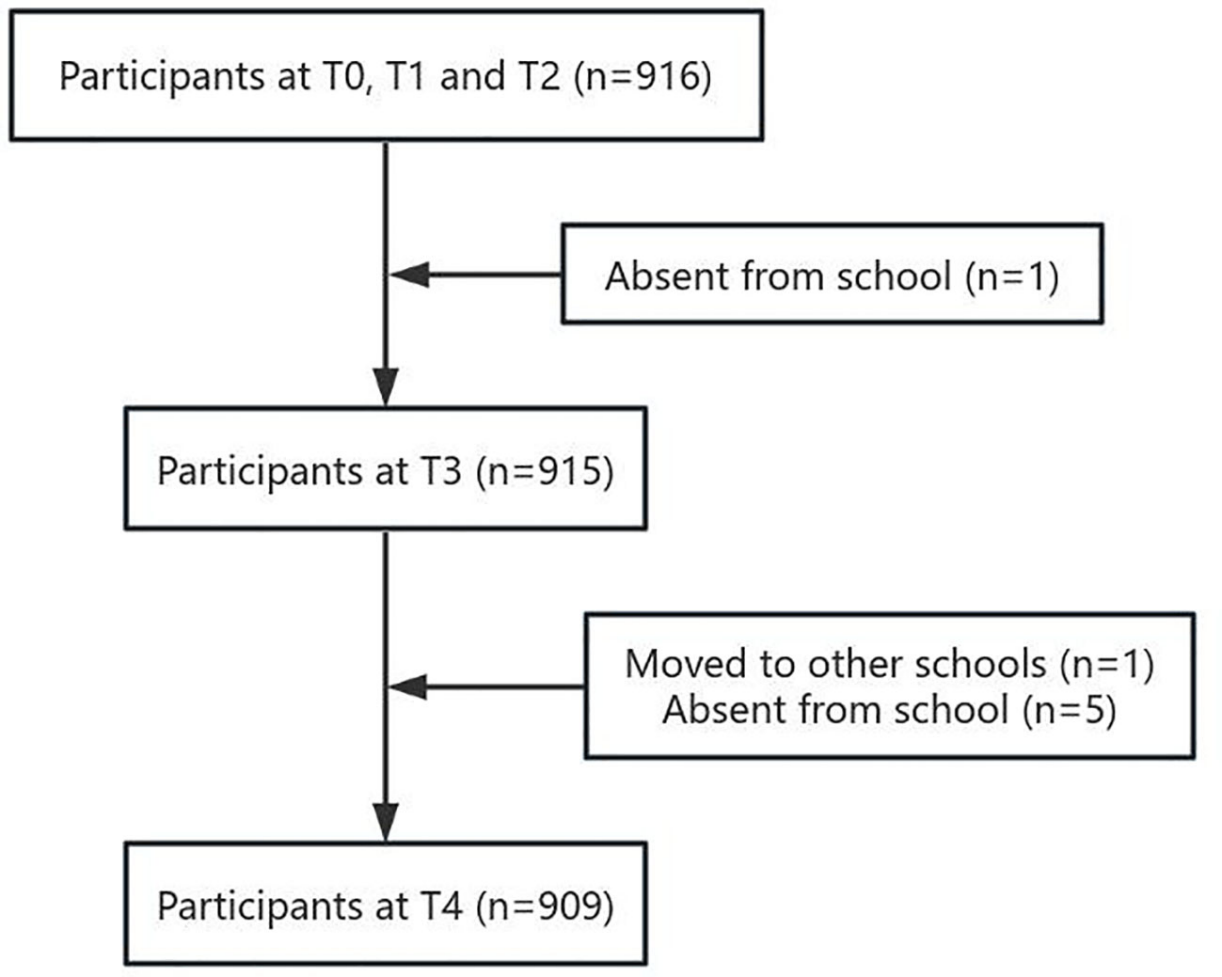
Flow chart of participants.

A baseline data sample (T0, n = 916) was collected in October 2018. Follow-up data were collected one month later (T1, n = 916), three months later (T2, n = 916), six months later (T3, n = 915), and 12 months later (T4, n = 909), as depicted in **Figure 1**. The independent sample t-test (28) revealed no significant differences in the scores of alienation towards mother (*t* = 1.343,*p* = 0.179) and alienation towards father (*t* = -0.300, *p* = 0.765) at T0 between the excluded subjects (n = 7) and the final sample (n = 909) who fully participated in this study.

Wave 1 (T0) had 428 (46.72%) girls and 488 (53.28%) boys (aged between 9 and 13 years old), indicating an almost equal gender proportion. The mean age of participants was 10.57 years old (S.D. = 1.03). Of the children, 196 (21.40%) children belonged to the “father absent” group, 41(4.48%) to the “mother absent” group, and 679 (74.12%) to the “both absent” group. See **Table 1** for more details.

**Table 1:** Demographic-social data of participants at baseline (T0).

Variables(N=916)	n(%)	Total IAP	t/F	Maternal form	t/F	Paternal form	t/F	Resilience	t/F	Subjective well-being	t/F
Age			1.74		1.39		1.45		0.91		0.97
9 years old	139(15.17)	33.63 ± 13.49		17.39 ± 7.45		16.24 ± 7.23		90.99 ± 17.13		20.5 ± 5.8	
10 years old	322(35.25)	31.26 ± 12.53		16.09 ± 7.58		15.16 ± 6.87		92.38 ± 15.2		20.68 ± 5.9	
11 years old	274(29.91)	33.42 ± 12.51		17.01 ± 7.3		16.41 ± 7.51		91.26 ± 17.07		21 ± 5.87	
12 years old	161(17.59	31.99 ± 11.94		16.74 ± 7.25		15.25 ± 6.48		93.12 ± 16.67		21.29 ± 5.9	
13 years old	20(2.18)	37.44 ± 12.94		19.94 ± 7.57		17.5 ± 9.08		86.28 ± 12.76		21.89 ± 4.97	
Gender			0.29		0.44		0.06		0.87		0.94
Male	488(53.28)	32.40 ± 12.41		16.65 ± 7.22		15.75 ± 7.23		92.31 ± 16.09		20.89 ± 5.77	
Female	428(46.72)	32.64 ± 12.85		16.86 ± 7.66		15.78 ± 6.99		91.37 ± 16.48		20.51 ± 6.28	
Grade			0.17		0.46		0.26		0.42		0.45
4	363(39.63)	32.81 ± 13.28		16.97 ± 7.67		15.84 ± 7.23		91.57 ± 16.14		20.79 ± 5.95	
5	292(31.88)	32.32 ± 12.52		16.42 ± 7.42		15.90 ± 7.39		91.54 ± 16.12		20.87 ± 5.9	
6	261(28.49)	32.31 ± 11.78		16.81 ± 7.12		15.49 ± 6.64		92.65 ± 16.65		20.42 ± 6.24	
Left-behind types			1.43		0.97		1.55		0.40		0.79
Mother absent	41(4.48)	31.88 ± 11.41		15.85 ± 5.57		16.02 ± 7.41		89.8 ± 15.94		21.78 ± 5.99	
Father absent	196(21.40)	31.22 ± 11.80		16.26 ± 7.29		14.97 ± 6.36		92.32 ± 16.66		20.48 ± 5.95	
Both absent	679(74.12)	32.92 ± 12.89		16.95 ± 7.56		15.97 ± 7.30		91.86 ± 16.19		20.71 ± 6.04	

### 2.2 Instruments

#### 2.2.1 Socio-demographic variables

We collected socio-demographic information using a self-designed demographic questionnaire, including gender, age, left-behind status, etc.

#### 2.2.2 The inventory of alienation towards parents

The IAP, designed in our previous work (29), assessed perceived alienation towards fathers and mothers in children aged 8–19. This scale consists of 18 items assessing two dimensions: maternal alienation (9 items, sample item: “I feel abandoned by my mother”) and paternal alienation (9 items, sample item: “I feel unable to communicate with my father”). Items are rated on a 5-point Likert scale from 1 (totally disagree) to 5 (totally agree), with higher scores meaning a higher level of alienation towards parents. This scale shows good reliability and validity in children aged 8–19 (20, 30). In this study, Cronbach’s alpha coefficients were 0.82 and 0.83 for paternal and maternal forms, respectively.

#### 2.2.3 The resilience scale for chinese adolescents

The RSCA assessed the participants’ psychological resilience (31). This scale consists of 28 items assessing five dimensions: goal concentration, interpersonal assistance, emotion regulation, positive perception, and family support. Items are rated on a 5-point Likert scale from 1 (totally disagree) to 5 (totally agree), with higher scores meaning greater resilience. The scale has shown good reliability and validity in Chinese children aged 9–16 (32, 33). In this study, Cronbach’s alpha coefficients ranged from 0.82–0.86 for total scale and sub-scales.

#### 2.2.4 The subjective happiness scale

The SHS was used to assess subjective well-being (34). This scale consists of 4 items (sample item: “In general, I consider myself”: 1-Not a very happy person…7-A very happy person). Responses were collected on a 7-point scale, with higher scores indicating greater happiness. In this study, Cronbach’s alpha coefficients ranged from 0.71–0.76.

### 2.3 Procedures

Before investigations, we obtained written informed consent from both children and their parents. The Human Research Ethics Committee of the Army Medical University approved the study (2021-28-03). The researchers explained the study to children and their parents in written and verbal. After obtaining informed consent, children completed the self-report questionnaires in their primary school classrooms at five time points (T0: baseline, T1: 1 month later, T2: 3 months later, T3: 6 months later, T4: 12 months later). After completing the fifth survey, the children were debriefed and received learning materials as incentives.

### 2.4 Data analysis

We applied an independent sample t-test and ANOVA to compare the differences of variables (alienation, resilience, and subjective well-being) at T0 among LBC using IBM SPSS 26.0. We further carried out a Pearson correlation analysis to observe their correlation. A hierarchical linear model (HLM) was utilized to observe the trend of five waves of subjective well-being in LBC and prediction from alienation. We carried out a multilevel linear model to explore the mediation of resilience in the relationship between alienation towards parents and LBC’s subjective well-being using HLM 6.08. All tests were two-tailed and performed at a significance level of *p* = 0.05.

HLM is a robust method of analyzing longitudinal data, allowing for handling missing data, non-fixed time intervals, and unequal error variance (35, 36). The HLM consists of a null model, an unconditional model, and a full model. 1) The null model, which included subjective well-being at five time points as the outcome variable without any predictor, was primarily utilized to assess the presence of a hierarchical structure in the developmental trajectory, i.e., whether the data is suitable for HLM analysis. 2) The unconditional model evaluated the linearity of the developmental trajectory of subjective well-being in LBC. According to the HLM analysis principle (35), time was considered an independent variable at Level 1, and the developmental trajectory of subjective well-being during five time points was considered the outcome variable. Then, an unconditional linear growth model was constructed to assess whether time significantly impacts the developmental trajectory of subjective well-being and whether it can further determine which variables of Level 1 are significantly affected by Level 2. If so, appropriate variables should be introduced in Level 2 (full model) for further analysis. 3) The full model utilized the Level 1 model to examine the impact of time (within-individual variable) on the developmental trajectory of subjective well-being. The Level 2 model investigated the influence of between-individual variables (e.g., alienation towards mother, alienation towards father, and resilience) on the developmental trajectory of subjective well-being.

## 3 Results

### 3.1 Baseline demographic-social description of participants

As shown in **Table 1**, there were no significant differences in the scores of alienation towards parents, resilience, and subjective well-being at T0 by age, gender, grade, or left behind type (*p*< 0.05).

As can be seen in **Table 2**, Pearson correlation analysis showed a positive correlation between resilience and subjective well-being at the same and different time points (*r* = 0.33 ~ 0.71, *p*< 0.001). In contrast, alienation towards parents was negatively correlated with them (-0.24~-0.44).

**Table 2:** Correlation analysis between alienation towards parents, resilience, and subjective well-being at five waves.

	M ± SD	1	2	3	4	5	6	7	8	9	10	11	12
1.T0 alienation towards mother	16.78 ± 7.44	1											
2.T0 alienation towards father	15.76 ± 7.11	0.50^***^	1										
3.T0 resilience	91.82 ± 16.29	-0.44^***^	-0.41^***^	1									
4.T1 resilience	93.45 ± 16.92	-0.34^***^	-0.33^***^	0.71^***^	1								
5.T2 resilience	92.42 ± 16.73	-0.30^***^	-0.27^***^	0.58^***^	0.68^***^	1							
6.T3 resilience	94.04 ± 17.23	-0.31^***^	-0.28^***^	0.61^***^	0.69^***^	0.71^***^	1						
7.T4 resilience	93.50 ± 16.62	-0.28^***^	-0.24^***^	0.49^***^	0.57^***^	0.60^***^	0.68^***^	1					
8.T0 subjective well-being	20.71 ± 6.02	-0.35^***^	-0.32^***^	0.53^***^	0.49^***^	0.40^***^	0.43^***^	0.38^***^	1				
9.T1 subjective well-being	20.95 ± 5.83	-0.32^***^	-0.32^***^	0.44^***^	0.52^***^	0.45^***^	0.49^***^	0.45^***^	0.61^***^	1			
10.T2 subjective well-being	21.38 ± 5.78	-0.27^***^	-0.26^***^	0.40^***^	0.44^***^	0.50^***^	0.47^***^	0.42^***^	0.55^***^	0.65^***^	1		
11.T3 subjective well-being	21.43 ± 5.62	-0.26^***^	-0.29^***^	0.35^***^	0.40^***^	0.42^***^	0.53^***^	0.43^***^	0.51^***^	0.61^***^	0.63^***^	1	
12.T4 subjective well-being	21.71 ± 5.66	-0.29^***^	-0.25^***^	0.33^***^	0.37^***^	0.38^***^	0.44^***^	0.50^***^	0.45^***^	0.54^***^	0.52^***^	0.62^***^	1

***p< 0.001.

### 3.2 Hierarchical linear models of subjective well-being

We utilized a hierarchical linear model to analyze the trend of subjective well-being in LBC and the predictive value of T0 alienation. Subsequently, we added age, gender, grade, and type of left-behind as control variables to the model for their potential influence on the dependent variables.

#### 3.2.1 Null model

Taking the LBC’s subjective well-being as the dependent variable, we established a null model (zero model). The results showed a significant variation in LBC’s subjective well-being (variance =19.00, *χ^2^ =* 6831.10, *df*=908, *p*<0.001), with the intra- and inter-group variances being 19.00 and 14.56, respectively. Afterward, the inter-class correlation (ICC) was found to be 0.57, showing significant variation between individual (37). Thus, it was necessary to build an unconditional growth model to explain further the reasons behind this difference (38, 39).

#### 3.2.2 Unconditional model

Consequently, an unconditional model was established, using the scores from the quintuple tracking of subjective well-being as the dependent variable and the time polynomial function as the independent variable. As shown in **Table 3**, across one-year tracking, LBC’s subjective well-being scores showed a significant linear growth trend (*γ_10_ =* 0.25, *p*<0.001).

**Table 3:** Estimated results of the unconditional model.

Fixed effects	Coefficient	SE	*t*	Random effects	Variance	df	χ^2^
Interceptγ_00_	20.49	0.22	94.96^***^	Interceptγ_0_	22.94	903	2406.37^***^
Slopeγ_10_	0.25	0.05	5.27^**^	Slopeγ_1_	0.72	903	1424.12^***^

**p<0.01, ***p<0.001.

#### 3.2.3 Full model

A full model of LBC’s subjective well-being was built, using the scores of alienation towards mother and father as predictive variables, while controlling for age, gender, grade, and types of left-behind. As shown in **Table 4**, the fixed effects showed that both alienation towards mother (*γ_05_* = -0.19, *p*< 0.001) and father (*γ_05_* = -0.19, *p*< 0.001) had significantly negative effects on subjective well-being. Grade significantly and negatively affected the rising rate of subjective well-being (*γ_13_* = -0.23, *p*< 0.01). The random effects indicated that alienation towards parents explained 33% (22.94–15.29/22.94 = 33%) of the total variance of subjective well-being.

**Table 4:** Estimated results of the full model.

Variables	Subjective Well-being		
Fixed effects	b-Weight	SE	*t*
Intercept			
Constantγ_00_	25.14	2.41	10.45^***^
Genderγ_01_	0.27	0.37	0.72
ageγ_02_	-0.04	0.30	-0.13
Gradeγ_03_	0.18	0.38	0.72
Left-behind typesγ_04_	0.28	0.34	0.47
Alienation towards motherγ_05_	-0.19	0.03	-6.74^***^
Alienation towards fatherγ_06_	-0.19	0.03	-6.26^***^
The slope			
Constantγ_10_	0.42	0.52	0.81
Genderγ_11_	0.01	0.08	0.09
Ageγ_12_	0.08	0.06	1.18
Gradeγ_13_	-0.23	0.08	-2.80^**^
Left-behind typesγ_14_	-0.10	0.07	-1.35
Alienation towards motherγ_15_	0.01	0.01	1.71
Alienation towards fatherγ_16_	0.01	0.01	2.13
Random effects	Variance	df	χ^2^
Level 1 (within individuals)r_0_	15.29	902	5714.63^***^
Level 2 (between individuals)r_1_	0.38	902	1432.71^***^

**p<0.01, ***p<0.001.

### 3.3 The mediating effect of resilience

**Figure 2:**
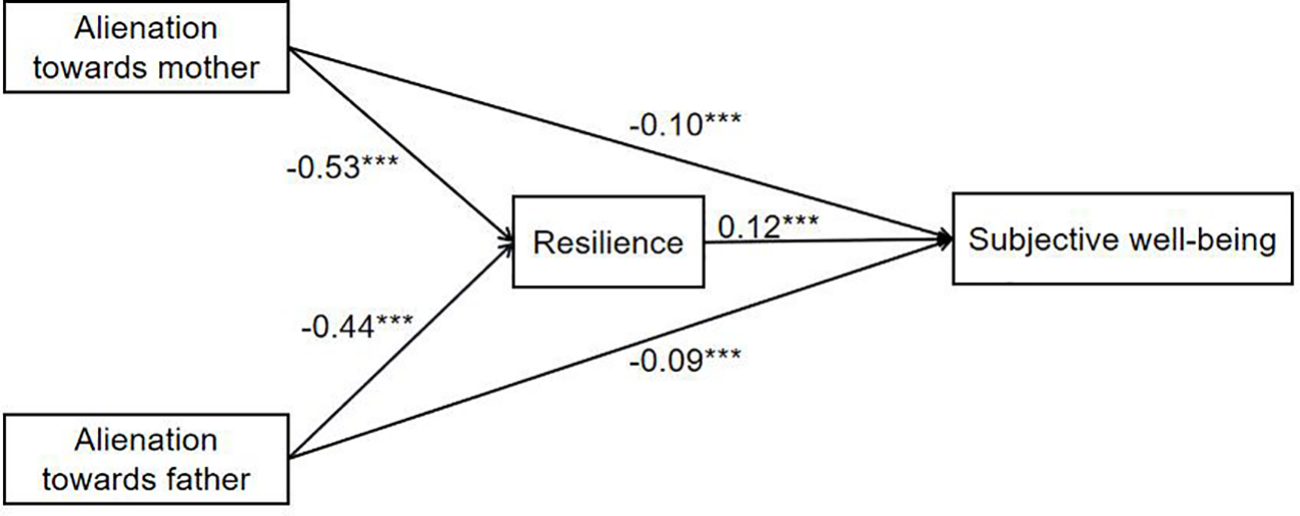
Mediation of resilience in the prediction of alienation towards parents on LBC’s subjective well-being. ***p<0.001.

We built a multilevel linear model to assess the mediating effect of resilience, with resilience and subjective well-being as dependent variables in Model 1 and Model 2, respectively. In Model 1, alienation towards both mother (γ_05_=-0.53, *p*<0.001) and father (γ_06_=-0.44, *p*<0.001) significantly negatively affected LBC’s resilience, indicating that alienation towards parents could negatively predict LBC’s resilience. When resilience was added into Model 2, the influence of resilience on subjective well-being was significant (γ_07_ = 0.12, *p*<0.001), and the effects of alienation towards both mother (γ_05_=-0.10, p<0.001) and father (γ_06_=-0.09, *p*<0.001) on subjective well-being were also significant. See **Table 5** and **Figure 2** for further details. These results indicated that resilience partially mediates the relationship between alienation towards parents and the LBC’s subjective well-being (40, 41).

**Table 5:** Multilevel linear model for the mediating effect of resilience.

Variables (intercepts of fixed effects)	Resilience(Model 1)			Subjective well-being(Model2)		
	b-Weight	SE	*t*	b-Weight	SE	*t*
Constantγ_00_	113.89	5.47	20.83^***^	12.71	1.67	7.62^***^
Genderγ_01_	-0.42	0.85	-0.48	0.34	0.24	1.40
Ageγ_02_	-0.07	0.69	-0.10	0.20	0.20	1.01
Gradeγ_03_	-0.42	0.87	-0.48	-0.46	0.25	-1.86
Left-behind typesγ_04_	-0.58	0.77	-0.75	0.05	0.22	0.23
Alienation towards motherγ_05_	-0.53	0.07	-8.16^***^	-0.10	0.02	-5.18^***^
Alienation towards fatherγ_06_	-0.44	0.07	-6.47^***^	-0.09	0.02	-4.77^***^
Resilienceγ_07_				0.12	0.01	23.59^***^

***p<0.001.

## 4 Discussions

The current study investigated the trend of subjective well-being, the predictive effect of alienation towards parents on the developmental trajectory of subjective well-being, and the potential mediation of resilience in rural LBC in China. There were three essential findings in this study. First, LBC’s subjective well-being increased over time. Second, alienation toward both parents negatively predicted the developmental trajectory of LBC’s subjective well-being. Third, resilience partially mediated the prediction of alienation towards parents on the LBC’s subjective well-being. Thus, to improve mental health status of LBC, their alienation towards parents and resilience level should be considered both in the family and at school.

Surprisingly, we found no significant differences in resilience, subjective well-being, and alienation towards parents across gender, grade, and types of left-behind. It could be explained by the general prevalence of the high alienation towards parents, low resilience, and low subjective well-being among all LBC, irrespective of their gender, grade, or types of left-behind. These findings stress the importance of universal attention to mental health issues among LBC. However, we should be very cautious when intending to conclude that there are no demographic differences in mental health levels among LBC, which warrants a larger sample size survey. Policymakers and healthcare professionals should develop comprehensive strategies to address the mental health needs of all LBC, regardless of their specific characteristics or demographics, e.g., offering resilience training in schools.

Correlation analysis results showed a significant negative correlation between both mother-child and father-child alienation scores and the LBC’s subjective well-being, which is consistent with the previous results (42). According to the ecosystem theory’s micro-system model (43), family is the most significant factor impacting children’s mental health and personal growth. The parent-child relationship, an important relationship in the family, directly affects children’s physical and mental development and subjective well-being (11, 44). For LBC, the integrity of family life has been severely compromised, which may lead to emotional alienation towards their parents due to spatial distance with parents, and further results in a reduction in LBC’s subjective well-being. Thus, families and schools must recognize the challenges LBC faces due to the absent parents and help mitigate workers to prevent these negative effects, e.g., strengthening bonds with children despite physical separation.

HLM showed a discernible tendency of linear increase in the subjective well-being scores of LBC. Regarding the level of LBC’s subjective well-being, most scholars believed that compared with non-LBC, LBC received less support from parents and lower subjective well-being during their growth (11, 45). However, some studies have found that staying at home or not of parents has little impact on rural children’s subjective well-being (3). Our study provides a more reasonable explanation of the two viewpoints from the perspective of the dynamic development of subjective well-being. Generally, due to the influence of the separation from parents, the LBC are deprived of intact parental care and show lower levels of subjective well-being. Thus, it is important for parents to realize the potential negative impact of their absence on their children and take necessary precautions. However, it is reassuring that the lower levels of well-being of LBC are not permanent. One of the reasons for this finding could be adaptive mechanisms (46) in children and their families over time. Alternatively, Factors contributing to the increase in LBC’s well-being might include the improved material circumstances accompanied by parents’ absence, children’s comprehension of parental leaving, and the relative expansion of personal space following parent-child separation (47). Moreover, individual differences exist in the growth rate of subjective well-being among LBC. It can be preliminarily inferred that the rising trend of LBC’s subjective well-being should result from various protective factors, e.g., lower grades. Therefore, caregivers should pay attention to the psychological dynamic changes in LBC, and schools should pay attention to LBC’s subjective well-being and proactively address any challenges they may encounter during this transitional period, e.g., by offering expressional opportunities about their feelings.

In addition, according to the multi-layer linear model results, both mother-child and father-child alienation could longitudinally predict the LBC’s subjective well-being. In other words, the effect of alienation towards parents on subjective well-being may be a long-term process. Although subjective well-being as a whole shows an upward trend, the improvements in adaptability or the psychological coping mechanisms of children themselves are insufficient to completely eliminate the negative effects of alienation towards parents on subjective well-being. Importantly, factors such as gender, grade, and types of left-behind do not significantly predict subjective well-being, which could be because, when children are in a left-behind status, individual factors’ influence on LBC’s subjective well-being is overshadowed by family factors. The parent-child relationship caused by being left-behind has become a primary influencing factor, which further suggests that LBC’s parents should keep in mind maintaining close bonds with their kids during extended absences. Regardless of the family’s specific circumstances, it is essential for absent parents to take on the responsibility of guardianship of their children, e.g., their children’s education, daily life, and feelings et al.

The multilevel linear model analysis of the intermediary role showed that psychological resilience significantly mediated the relationship between alienation towards parents and LBC’s subjective well-being, which suggests that alienation towards parents can not only directly affect and predict LBC’s subjective well-being levels but also indirectly affect them by psychological resilience. We found that the psychological resilience level of rural LBC in this study was much lower than that of 5976 urban pupils in Zou’s study (48). Based on the theory of psychological resilience development, a close relationship between children and their parents in high-risk situations is more likely to make them develop a higher level of psychological resilience (49). We can regard parent-child separation caused by parents’ absence as a high-risk situation for parent-child relationship. A high level of alienation towards parents, indicating a failure to establish a harmonious parent-child relationship during parents’ leaving, is naturally not conducive to LBC’s psychological resilience development. However, as our study’s participants are primary school children in rural areas, we cannot rule out the impact of education level, growth environment, and other influencing factors on psychological resilience. The results suggested that schools and other educational agencies could take psychological flexibility as a starting point to promote the mental health of LBC by strengthening psychological resilience-related training and counseling.

Limitations: First, we used a convenient sampling, which might affect the sample’s representativeness. Second, the follow-up period was only 12 months instead of a longer time. Third, as a comparison, we did not recruit non-LBC participants for comparison, whose mental health development trends might differ from LBC. However, based on a follow-up design, our study investigated the trend of alienation towards parents in LBC and the prediction from alienation and the mediating role through psychological resilience, which provides reliable evidence for schools and government bodies to formulate policies and psychological interventions.

To sum up, our study indicates that LBC exhibit lower levels of subjective well-being, which significantly increases over time. Alienation towards parents negatively predicts LBC’s subjective well-being, with psychological resilience acting as a mediator between them. Strategies aimed at promoting LBC’s mental health should focus on reducing their alienation towards parents and improving their psychological resilience. Moreover, we encourage LBC’s parents to strengthen emotional communication with their children, and the government can also optimize rural migrant work in various ways.

## Data availability statement

The raw data supporting the conclusions of this article will be made available by the authors, without undue reservation.

## Ethics statement

The studies involving humans were approved by the Human Research Ethics Committee, Army Medical University (2021-28-03). The studies were conducted in accordance with the local legislation and institutional requirements. Written informed consent for participation in this study was provided by the participants’ legal guardians/next of kin. Written informed consent was obtained from the minor(s)’ legal guardian/next of kin for the publication of any potentially identifiable images or data included in this article.

## Author contributions

MZ: Formal analysis, Investigation, Writing – original draft, Writing – review & editing. XS: Investigation, Writing – original draft, Writing – review & editing. CC: Formal analysis, Writing – review & editing. JT: Writing – review & editing. XR: Formal analysis, Writing – original draft. QD: Conceptualization, Data curation, Formal analysis, Funding acquisition, Supervision, Writing – review & editing.

## References

[1] Fan Z Wu Y. Relationship between parent-child relationship, loneliness and depression among the left-behind rural children: gratitude as a mediator and a moderator. psychol Dev Educ. (2020) 06(36):734–42. doi: 10.16187/j.cnki.issn1001-4918.2020.06.12

[2] Lyu L Mei Z Li R. Status and changes of the rural children left behind in China: 2010–-2020 (in Chinese). Popul Res. (2024) 1:103–17.

[3] Su S Li X Lin D Xu X Zhu M. Psychological adjustment among left-behind children in rural China: the role of parental migration and parent-child communication. Child Care Health Dev. (2013) 39:162–70. doi: 10.1111/j.1365-2214.2012.01400.x 22708901

[4] Gong C. China’s Population Development Presents New Characteristics and New Trend[EB/OL]. [13 May]. Available online at: http://finance.people.com.cn/n1/2021/0513/c1004–32101889.html.

[5] Li R Liu H Zhang Q. Practice and experience of the 7th population census: how to ensure and improve data quality? (in Chinese) Popul Res. (2021) 5(45):26–31.

[6] Nilsen S Bøe T. Divorce and family structure in Norway – Associations to adolescent mental healthSondre Nilsen. Eur J Public Health. (2016) 26:89. doi: 10.1093/eurpub/ckw166.041

[7] Aske A Carsten BP Pearl LHM Carr MJ Webb RT. Self-harm risk between adolescence and midlife in people who experienced separation from one or both parents during childhood. J Affect Disord. (2017) 208:582–9. doi: 10.1016/j.jad.2016.10.023 PMC5754328 27802894

[8] Liu Z Li X Xiaojia G. Left too early: the effects of age at separation from parents on Chinese rural children’s symptoms of anxiety and depression. Am J Public Health. (2009) 99:2049–54. doi: 10.2105/AJPH.2008.150474 PMC2759782 19762669

[9] Ed D Robert AE Randy JL Griffin S. The satisfaction with life scale. J Pers Assess. (1985) 49:71–5. 10.1207/s15327752jpa4901_13 16367493

[10] Li Z S M. A survey of subjective well-being of rural college students and analysis of related factor. (in Chinese). Agric Archaeol. (2007) 2007(6):250–2.

[11] Fan Z Fan X. Parent-child relationship and general well-being among the left-behind rural children: psychological capital as a mediator and pocket money as a moderator. Chin J Clin Psychol. (2020) 28:624–7. doi: 10.16128/j.cnki.1005-3611.2020.03.039

[12] Chen L Z L S J. The effect of parenthood of left-behind children in rural areas on their subjective well-being. (in Chinese). Chin J Special Educ. (2009) 2009(3):8–12.

[13] Yan L X L. Rural left-behind children’s parent-child attachment, teacher-student relationship and subjective well-being under fathering absence. Chin J Clin Psychol. (2013) 3:493–6. doi: 10.16128/j.cnki.1005-3611.2013.03.012

[14] Chai H-Y S X N G. Effects of parents-adolescents relations and friendship quality on subjective well-being: indirect effect model and gender differences. Chin J Clin Psychol. (2016) 3:531–4. doi: 10.16128/j.cnki.1005-3611.2016.03.033

[15] Wen-guang SGH. Parent-adolescent conflict: A retrospective and prospective view (in Chinese). J Nanjing Normal University (Social Sci). (2011) 2011(04):105–10.

[16] Wu M L Z L L. Parent-child relationship impact on children’s mental development. (in Chinese). J Beijing Normal Univ. (2016) 2016(5):55–63.

[17] Farkas MM. An introduction to parental alienation syndrome. J Psychosocial Nurs Ment Health Serv. (2011) 49:20–6. doi: 10.3928/02793695-20110302-02 21410089

[18] Bernet W Baker AJ Verrocchio MC. Symptom Checklist-90-Revised scores in adult children exposed to alienating behaviors: an Italian sample. J Forensic Sci. (2015) 60:357–62. doi: 10.1111/1556-4029.12681 25613416

[19] Sun D Q J. Does left-behind estrange the relationship between children and parents? Empirical evidence from CEPS. (in Chinese). South China Popul. (2020) 45:33–45.

[20] Sun X S P S S. Alienation towards parents and its influential factors in rural left-behind children of Chongqing. China J Health Psychol. (2020) 4:562–9. doi: 10.13342/j.cnki.cjhp.2020.04.019

[21] Sher L. Parental alienation: the impact on men’s mental health. Int J Adolesc Med Health. (2015) 29:1–5. doi: 10.1515/ijamh-2015-0083 26565536

[22] Luthar. Resilience and vulnerability[M]. Cambridge University Press (2003) isbn: 0521807018 (hb), 0521001617 (pbk.).

[23] Ronald ME Michael SK. Physician resilience: what it means, why it matters, and how to promote it. Acad Med. (2013) 88:301–3. doi: 10.1097/ACM.0b013e318280cff0 23442430

[24] Chai X-Y G H L D. The emotion regulation strategies and the psychological well-being among migrant children in China: the roles of self-esteem and resilience. J psychol Sci. (2018) 1:71–7. doi: 10.16719/j.cnki.1671-6981.20180111

[25] Feng X-Z Z L S H. Study of the relationship between resilience and subjective well-being of junior high school left-behind children. (in Chinese). Chin J Child Health Care. (2016) 6:573–5.

[26] Hongsheng L Lige L Xiaoyi J. The impact of parental remote migration and parent-child relation types on the psychological resilience of rural left-behind children in China. Int J Environ Res Public Health. (2020) 17:5388. doi: 10.3390/ijerph17155388 32726979 PMC7432675

[27] Aphichat C Aksarapak L Aree J Kathleen F. An analysis of the resilience process: The stimulus of mental strength and the role of community and family support amidst the civil violence in Thailand. Curr Psychol. (2020) 41(8):1–7. doi: 10.1007/s12144-020-01002-w

[28] Banda G. A brief review of independent, dependent and one sample t-test. Int J Appl Mathematics Theor Phys. (2018) 4:50. doi: 10.11648/j.ijamtp.20180402.13

[29] Dai Q Yang G Hu C Wang L Liu K Guang Y. The alienation of affection toward parents and influential factors in Chinese left-behind children. Eur Psychiatry. (2017) 39:114–22. doi: 10.1016/j.eurpsy.2016.07.008 28006677

[30] Sun X-X R H S P. Study on the alienation towards parents and its mediating effect between life-event and depression in rural left-behind children. (in Chinese). Chongqing Med. (2020) 20:8.

[31] Hu Y-Q Gan Y. Development and psychometric validity of the resilience scale for Chinese adolescents. (in Chinese). Acta Psychologica Sin. 2008(8):902–12.

[32] Diao H Ran M Yang J-W Yang L-J Li T Jin F. Moderating role of peer attachment in the relationship between adolescent knowledge-attitudepractice and psychological resilience. in Chinese). J Shanghai Jiao Tong Univ (Medical Sci). (2020) 8:1120–5.

[33] Yang W H D H. Relationship between adolescent knowledge-attitude-practice and resilience in left-behind children. (in Chinese). Chin J Sch Health. (2020) 7:1032–5.

[34] Leppe S L A H. A measure of subjective happiness: preliminary reliability and construct validation. Soc Indic Res. (1999) 46:137–55.

[35] Yeh PH Gazdzinski S Durazzo TC Sjostrand K Meyerhoff DJ. Hierarchical linear modeling (HLM) of longitudinal brain structural and cognitive changes in alcohol-dependent individuals during sobriety. Drug Alcohol Depend. (2007) 91:195–204. doi: 10.1016/j.drugalcdep.2007.05.027 17644276

[36] Gai X-S Z X. The application of multilevel model in longitudinal research. psychol Sci. (2005) 2:429–31. doi: 10.16719/j.cnki.1671-6981.2005.02.043

[37] James LR. Aggregation bias in estimates of perceptual agreement. J Appl Psychol. (1982) 67:219–29. doi: 10.1037/0021-9010.67.2.219

[38] Wu YW Wooldridge PJ. The impact of centering first-level predictors on individual and contextual effects in multilevel data analysis. Nurs Res. (2005) 54:212–6. doi: 10.1097/00006199-200505000-00009 15897797

[39] Chen YX. Multi-layer linear modelling of social adaptation development trends of college students. (in Chinese). China J Health Psychol. (2024) 2:295–301.

[40] Fang J Zhang M Chiou H-J. Multilevel mediation based on hierarchical linear model. Adv psychol Sci. (2010) 8:1329–38.

[41] Ao HJZD. The developmental trajectory of student teachers’ Professional identity in the early stages of teaching practice and its relationship with proactive personality: A longitudinal study. psychol Dev Educ. (2023) 5:40–7.

[42] Qin X Sun X Zhang M Chen B Xie F Chen Z. Life-events mediate the prediction of parental alienation on depression in rural left-behind children: A longitudinal study. Front Psychiatry. (2022) 13:864751. doi: 10.3389/fpsyt.2022.864751 35782429 PMC9247398

[43] Liu H J X C Z. The mental resilience of rural left-behind children: population differentiation, influencing factors, and intervention measures. (in Chinese). J Southwest Jiaotong University (Social Sci). (2019) 2019(1):31–7.

[44] Ye Y Duo BLY. Study of parents-child relationship and it's influence on child development at home and abroad. (in Chinese). J Fujian Normal University (Philosophy Soc Sci Edition). 2002:130–6.

[45] Suo HKLY. The social adjustment of left-behind children in rural Chian: A propensity score analysis. psychol Dev Educ. (2014) 6:88–97. doi: 10.16187/j.cnki.issn1001-4918.2014.06.011

[46] Zhao X Fu F Zhou L. The mediating mechanism between psychological resilience and mental health among left-behind children in China. Children Youth Serv Rev. (2020) 110:104681–6. doi: 10.1016/j.childyouth.2019.104686

[47] Aiqin L. An effective method of producing language and thinking for deaf people. Chin J Special Educ. (2009) 2009(3):8–12.

[48] Rong ZYYY. Influence of psychological resilience on cognitive bias towards school violence among primary school students in Luzhou. Chin J School Health. (2019) 12:1842–5. doi: 10.16835/j.cnki.1000-9817.2019.12.021

[49] Emmy EW. Resilience in development. Curr Dir psychol Sci. (1995) 4:81–4. doi: 10.1111/1467-8721.ep10772327

